# Predictive value of baseline metabolic tumor volume in early-stage favorable Hodgkin Lymphoma – Data from the prospective, multicenter phase III HD16 trial

**DOI:** 10.1186/s12885-022-09758-z

**Published:** 2022-06-18

**Authors:** Lutz van Heek, Colin Stuka, Helen Kaul, Horst Müller, Jasmin Mettler, Felicitas Hitz, Christian Baues, Michael Fuchs, Peter Borchmann, Andreas Engert, Markus Dietlein, Conrad-Amadeus Voltin, Carsten Kobe

**Affiliations:** 1 Department of Nuclear Medicine, Faculty of Medicine and University Hospital Cologne, University of Cologne, Kerpener Straße 62, 50937 Cologne, Germany; 2 First Department of Internal Medicine and German Hodgkin Study Group (GHSG), Center for Integrated Oncology Aachen – Bonn – Cologne – Düsseldorf (CIO ABCD), Faculty of Medicine and University Hospital Cologne, University of Cologne, Cologne, Germany; 3 Swiss Group for Clinical Cancer Research, Bern, Switzerland; 4 Department of Medical Oncology and Haematology, Kantonsspital St.Gallen, St. Gallen, Switzerland; 5 Department of Radiation Oncology and Cyberknife Center, Faculty of Medicine and UniversityHospital Cologne, University of Cologne, Cologne, Germany

**Keywords:** Hodgkin lymphoma, Metabolic tumor volume, FDG PET, Response prediction

## Abstract

**Background:**

^18^F -fluorodeoxyglucose (FDG) positron emission tomography (PET) plays an important role in the staging and response assessment of lymphoma patients. Our aim was to explore the predictive relevance of metabolic tumor volume (MTV) and total lesion glycolysis (TLG) in patients with early stage Hodgkin lymphoma treated within the German Hodgkin Study Group HD16 trial.

**Methods:**

^18^F-FDG PET/CT images were available for MTV and TLG analysis in 107 cases from the HD16 trial. We calculated MTV and TLG using three different threshold methods (SUV_4.0,_ SUV_41%_ and SUV_140%L_), and then performed receiver-operating-characteristic analysis to assess the predictive impact of these parameters in predicting an adequate therapy response with PET negativity after 2 cycles of chemotherapy.

**Results:**

All three threshold methods analyzed for MTV and TLG calculation showed a positive correlation with the PET response after 2 cycles chemotherapy. The largest area under the curve (AUC) was observed using the fixed threshold of SUV_4.0_ for MTV- calculation (AUC 0.69 [95% CI 0.55–0.83]) and for TLG-calculation (AUC 0.69 [0.55–0.82]). The calculations for MTV and TLG with a relative threshold showed a lower AUC: using SUV_140%L_ AUCs of 0.66 [0.53–0.80] for MTV and 0.67 for TLG [0.54–0.81]) were observed, while with SUV_41%_ an AUC of 0.61 [0.45–0.76] for MTV, and an AUC 0.64 [0.49–0.80]) for TLG were seen.

**Conclusions:**

MTV and TLG do have a predictive value after two cycles ABVD in early stage Hodgkin lymphoma, particularly when using the fixed threshold of SUV_4.0_ for MTV and TLG calculation.

**Trial registration:**

ClinicalTrials.gov NCT00736320.

## Background

Over the past few decades, Hodgkin lymphoma has become an effectively treatable malignancy with excellent long-term disease-free survival [1]. Nowadays, more than 90% of early-stage patients can be cured through first-line therapy, consisting of brief chemotherapy followed by 20 Gy consolidative irradiation [2–5]. Furthermore, treatment insensitivity in connection with both chemo- and radiotherapy has decreased considerably for patients with Hodgkin lymphoma at different tumor stages [6–8].

Since the introduction of ^18^F -fluorodeoxglucose (FDG) positron emission tomography (PET) into the management of many oncological diseases, it has taken on a major role in the staging and response assessment of lymphoma patients [9–12]. It has been shown that radiotherapy can safely be omitted in patients with PET-negative residual tissue after effective first-line chemotherapy for advanced-stage Hodgkin lymphoma [13]. Furthermore in advanced Hodgkin lymphoma, chemotherapy can be reduced to a total of 4 cycles eBEACOPP (Bleomycin, Etoposide, Doxorubicin, Cyclophosphamide, Vincristine, Procarbazine and Prednisone in escalated doses) if the PET is negative after 2 cycles [14]. For patients with early-stage unfavorable Hodgkin lymphoma, the HD17 trial has shown that radiotherapy can be omitted for PET-negative patients after effective chemotherapy without any clinically relevant loss of efficacy [15]. However, in early stage favorable Hodgkin lymphoma, three different randomized trials (H10, HD16 and RAPID) have shown that omitting radiotherapy in patients who are PET-negative after ABVD (doxorubicin, bleomycin, vinblastine and dacarbazine) chemotherapy is associated with a relevant loss of tumor control and increased number of relapses [6, 16, 17]. As the role of PET for individual tailoring of treatment is limited after 2 cycles of ABVD in early-stage favorable Hodgkin lymphoma, additional prognostic factors are needed urgently. Accordingly, we performed an analysis of metabolic tumor volume (MTV) and total lesion glycolysis (TLG) derived from PET at staging as potentially useful predictive factors, in early-stage Hodgkin lymphoma.

## Methods

### Study cohort

From November 2009 through December 2015, the prospective, multicenter phase III trial HD16 recruited a total of 1,150 therapy-naive Hodgkin lymphoma patients, aged 18 to 75 years. HD16 included patients in clinical stage I or II without risk factors such as three or more involved nodal areas, large mediastinal mass (≥ 1/3 of the maximal thoracic diameter as measured on chest X-ray), extra-nodal disease or elevated erythrocyte sedimentation rate (≥ 50 mm/h for patients without B symptoms and ≥ 30 mm/h in case of B symptoms).

**Fig. 1:**
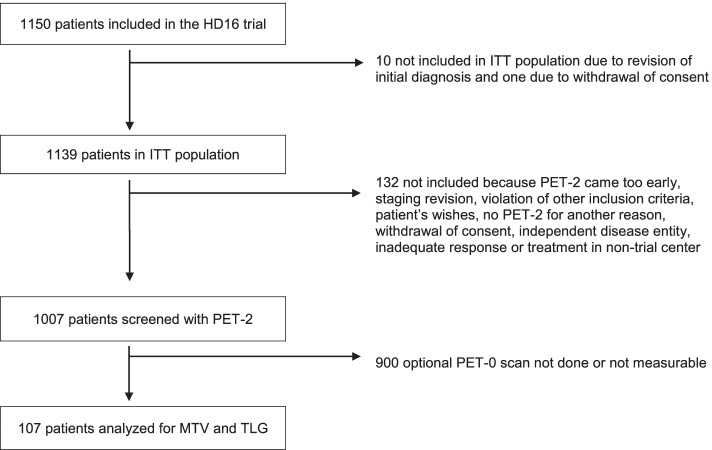
Flow chart. 1150 patients included in the HD16 trial. Baseline ^18^F-FDG PET was centrally reviewed in 107 patients with quantitative analyses. Abbreviations: ITT, intention to treat. HL, Hodgkin lymphoma. MTV, metabolic tumor volume. TLG, total lesion glycolysis

In HD16, individuals were randomly assigned either to standard combined-modality treatment including 2 cycles of ABVD followed by PET (PET-2) and consolidating radiotherapy irrespective of PET-2 result, or to the experimental arm where irradiation was omitted in cases of PET negativity after 2 cycles of ABVD. In HD-16 PET-2 was mandatory for all patients, while a staging PET before start of treatment was not a mandatory part of the protocol. The PET scans were performed according to the respective national guidelines, which did not include SUV harmonization. Accordingly, our analysis set consisted of those 107 individuals with baseline PET (PET-0) images available to the central review panel for quantitative assessment. (Fig. 1).

The HD16 trial was approved by the responsible ethics committees and was conducted according to the Declaration of Helsinki, in compliance with the Good Clinical Practice guidelines of the International Conference on Harmonization. All patients provided written informed consent before participation.

### Image analysis

Baseline MTV and TLG were calculated in all baseline PET scans available for quantitative analyses, using the PET/CT Viewer in FIJI (ImageJ). First, maximal standardized uptake value (SUVmax) for the liver was obtained from a spherical 3-cm volume of interest (VOI) in the right liver lobe. Following that, SUVmax was estimated within all tumor sites with increased F-FDG uptake. Manual corrections were performed in cases where non-lymphoma tissue was included in the automatic calculation.

MTV calculations were then performed using the following thresholding methods:

- 1. 41% of the SUVmax within the respective lymphoma site (MTV41%),
- 2. a fixed SUV of 4.0 (MTV4.0),
- 3. 140% of the SUVmax of liver background (MTV140%L).

Within these MTVs, SUVmean was estimated and TLG was calculated as the sum of supra-threshold voxels of all lymphoma lesions multiplied by SUVmean within the respective MTV as follows:

- 1. Sum of MTV_41%_ multiplied by SUVmean (TLG_41%_),
- 2. Sum of MTV_4.0_ multiplied by SUVmean (TLG_4.0_),
- 3. Sum of MTV_140%L_ multiplied by SUVmean (TLG_140%L_),

### Statistical evaluation

Patient characteristics and PET-2 response data were obtained from the study database. All data were analyzed descriptively. MTV and TLG distributions were visualized in histograms. The correlation of the different thresholding methods was assessed by Pearson product moment correlation coefficients. Receiver operating characteristic (ROC) analysis was performed to evaluate baseline MTV and TLG as predictors of PET-2 response, using the liver as cutoff for PET positivity (Deauville score 4) [18, 19]. Additionally, p-values resulting from logistic regressions on log-transformed data are reported to explore and quantify the predictive value of MTV and TLG on PET-2 positivity. All statistical computations were performed using SAS 9.4 (SAS Institute, Cary, NC, USA).

## Results

### Patients

Of the 1,139 patients from the intention-to-treat population of the HD16 study, 107 with available PET-0 were eligible for the present analysis. Characteristics of eligible and non-eligible patients are shown in Table 1. Among the 4 participating countries, the proportion of patients receiving a PET-0 scan was lowest in Germany. Other characteristics were similar in patients with and without PET-0. In PET-2, 16 (15%) of the patients examined were positive (Deauville Score 4) while 91 (85%) were negative (Deauville Score < 4).

**Table 1:** Patient characteristics

		**MTV and TLG measured**	**PET-0 not done or not measurable**
		*N* = 107	*N* = 1032
**Age**	Median (range)	36 (18–75)	38 (18–75)
**Sex**	Female	44 (41%)	441 (43%)
	Male	63 (59%)	591 (57%)
**Country**	Germany	82 (77%)	935 (91%)
	Switzerland	13 (12%)	49 (5%)
	Austria	7 (7%)	34 (3%)
	Netherlands	5 (5%)	14 (1%)
**Performance status**	ECOG = 0	100 (94%)	948 (92%)
	ECOG = 1	7 (6%)	82 (8%)
	ECOG = 2	-	1 (< 1%)
**Ann Arbor stage**	IA	31 (29%)	278 (27%)
	IB	4 (4%)	42 (4%)
	IIA	69 (65%)	656 (64%)
	IIB	3 (3%)	56 (5%)
**Treatment group**	Standard CMT	59 (55%)	514 (50%)
	PET-stratified	48 (45%)	518 (50%)
**PET-2 result**	DS1	49 (46%)	463/932 (50%)
	DS2	13 (12%)	159/932 (17%)
	DS3	29 (27%)	199/932 (21%)
	DS4	16 (15%)	111/932 (12%)
**Failure in terms of PFS**	No	99 (93%)	943 (91%)
	Yes	8 (7%)	89 (9%)

### MTV

**Fig. 2:**
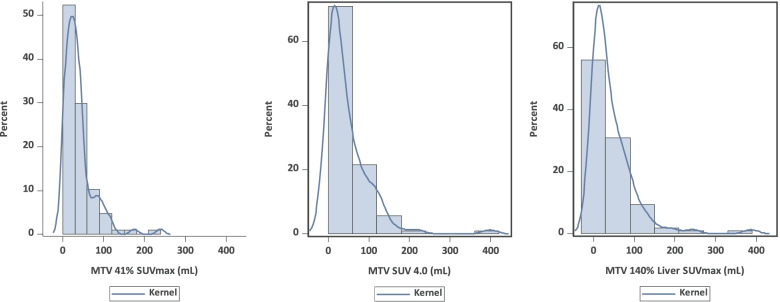
Histograms of MTV distribution assessed by different thresholding methods. MTV was obtained using the following thresholds: 41% of the SUV_max_ within the respective lymphoma site (MTV_41%_), a fixed SUV of 4.0 (MTV_4.0_), and 140% of the SUV_max_ of liver background MTV_140%L_. Abbreviations: MTV, metabolic tumor volume

Histograms of the MTV distributions are presented in Fig. 2 and show a right-skewed distribution for all thresholding methods. The median values of MTV_41%_, MTV_4.0_, and MTV_140%L_ were 28.7 mL (range, 0.9 – 238.9), 27.4 mL (range, 0.34 – 397.8) and 24.4 mL (range, 0.1 – 386.4). The correlation between the two relative threshold methods was 0.70, while the correlations with the absolute thresholding method (MTV_4.0_) were 0.70 for MTV_41%_ and 0.94 for MTV_140%L_, respectively. Low correlation of MTV_41%_ might have been caused by the relatively lower volume compared to MTV_4.0_ and MTV_140L_ in the case of a higher SUV_max_.

### TLG

**Fig. 3:**
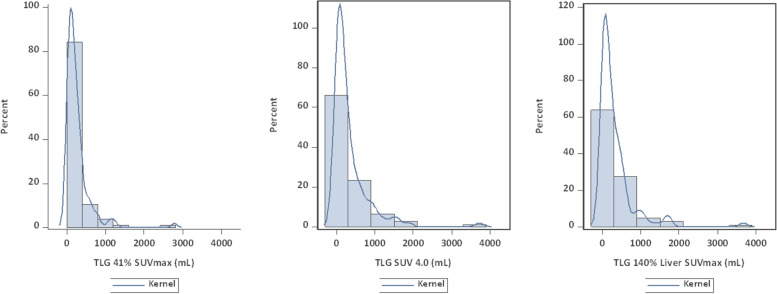
Histograms of TLG distribution assessed by different thresholding methods. TLG was obtained using the following thresholds: 41% of the SUV_max_ within the respective lymphoma site (TLG_41%_), a fixed SUV of 4.0 (TLG_4.0_), and 140% of the SUV_max_ of liver background (TLG_140%L_). Abbreviations: TLG, total lesion glycolysis

Histograms of the TLG distributions are presented in Fig. 3 and show a right-skewed distribution for all thresholding methods. The SUVmean for MTV_41%_ was 6.8 (± 3.2), MTV_4.0_ 6.3 (± 1.8), and MTV_140%L_ 6.6 (± 2.0). The median of TLG_41%,_ TLG_4.0_, and TLG_140%L_ were 162.0 (range, 5.8 – 2771.5), 160.5 (range, 1.4 – 3715.3) and 147 mL (range, 0.2 – 3670.8). The correlation between the two relative threshold methods was 0.94, while the correlations with the absolute thresholding method (TLG_4.0_) were 0.92 for TLG_41%_ and 0.97 for TLG_140%L_, respectively.

### Effect of MTV on PET-2 positivity

**Fig. 4:**
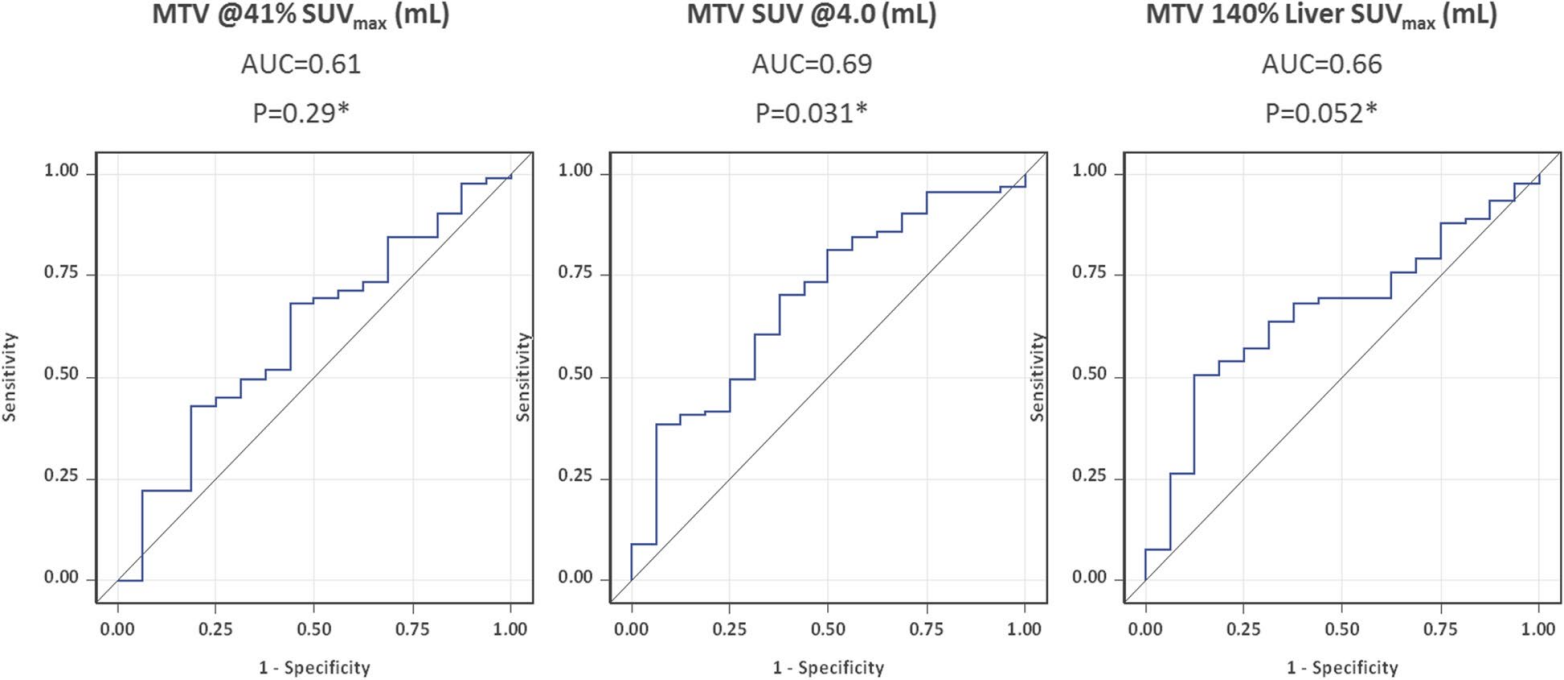
ROC curves of MTV distribution assessed by different thresholding methods for PET response after two cycles of ABVD (16/107 = 15% PET-2-positive patients with Deauville score 4). MTV was obtained using the following thresholds: 41% of the SUV_max_ within the respective lymphoma site (MTV_41%_) a fixed SUV of 4.0 (MTV4.0) and 140% of liver background (MTV_140%L_). Abbreviations: MTV, metabolic tumor volume. AUC, area under the curve

The ROC curves for PET response after two cycles of ABVD were derived from MTV using different thresholding methods, and are displayed in Fig. 4. Areas under the curve (AUC) for MTV_41%_, MTV_4.0_, and MTV_140%L_ were 0.61 (95% CI 0.45–0.76, *P*_*logist*_ = 0.29), 0.69 (0.55–0.83, *P*_*logist*_ = 0.031), and 0.66 (0.53–0.80, *P*_*logist*_ = 0.052).

### Effect of TLG on PET-2 positivity

**Fig. 5:**
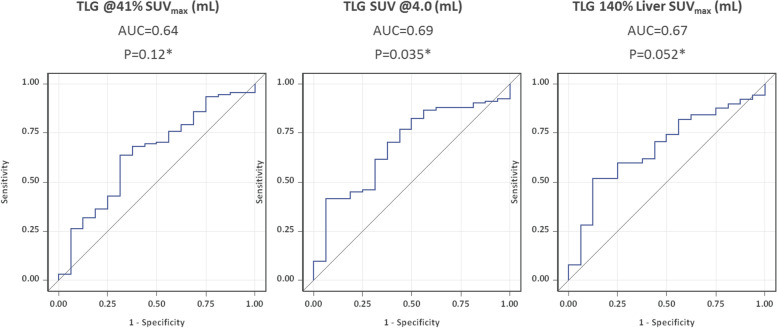
ROC curves of TLG distribution assessed by different thresholding methods for PET response after two cycles of ABVD (16/107 = 15% PET-2-positive patients with Deauville score 4). TLG was obtained using the following thresholds: 41% of the SUV_max_ within the respective lymphoma site (TLG_41%_) a fixed SUV of 4.0 (TLG4.0) and 140% of the SUV_max_ of liver background (TLG_140%L_). Abbreviations: TLG, total lesion glycolysis. AUC, area under the curve

The ROC curves for PET response after two cycles of ABVD were derived from TLG using different thresholding methods, and are displayed in Fig. 5. AUC for TLG_41%_, TLG_4.0_ and TLG_140%L_ were 0.64 (95% CI 0.49–0.80, *P*_*logist*_ = 0.12), 0.69 (0.55–0.82, *P*_*logist*_ = 0.035), and 0.67 (0.54–0.81, *P*_*logist*_ = 0.052).

## Discussion

The following results emerge from our analysis of 107 patients with early-stage favorable Hodgkin lymphoma: All three methods used for calculating MTV and TLG show a moderate predictive impact with regard to PET response after 2 cycles of ABVD in early-stage favorable Hodgkin lymphoma. Both the calculations of MTV and TLG using SUV 4.0 as fixed threshold, showed a small advantage, as compared to the other methods used.

Various studies have indicated the prognostic potential of baseline MTV in Hodgkin lymphoma patients [19–26]. Akhtari and colleagues showed that MTV and TLG could help predict worse outcome in 267 patients with early-stage Hodgkin lymphoma who received combined standard modality treatment. Furthermore, two distinct categories can be discerned from MTV or TLG: low and high disease burdens [20]. In a study of 59 patients with Hodgkin lymphoma treated with anthracycline-based chemotherapy, Kanoun and colleagues highlighted a possible division into two risk groups with regard to long-term success on the basis of the MTV and the metabolic signature [21]. Cottereau and colleagues showed baseline MTV to be a strong prognostic factor in 258 patients with early-stage Hodgkin lymphoma who received standard combined modality treatment. This collective of patients could also be divided into two risk groups based on the MTV [22]. In another group of 127 patients, Song and colleagues showed that MTV can be a prognostic factor and can also usefully influence selection of the necessary therapy regimen [23]. In 65 patients with a relapsed or refractory Hodgkin, Moskowitz and colleagues found MTV to be a very strong prognostic factor and one that can also improve the predictive value of PET before autologous stem cell transplantation [24]. In 310 patients with advanced Hodgkin lymphoma, Mettler and colleagues have shown that the MTV can predict patient response after two cycles of eBEACOPP, regardless of the method used to determine it [25]. The receiver-operating-characteristic curves in their study did not point to any unique cut-offs, but indicated a wide range of possible cut-offs [25], as we have also observed here in the present work. Analyzing a group of 140 DLBCL patients, Kim and colleagues show that the metabolic tumor burden expressed as TLG can be a prognostic factor for survival after R-CHOP [26]. All these studies are in line with our finding that initial MTV and TLG are parameters of additional use for response prediction.

Here, it should be noted that there is as yet no standardized procedure for measuring MTV and TLG [27]. A variety of methods and software platforms are currently in use for MTV and TLG calculation. The use of algorithms with fixed-threshold or relative-threshold values and adaptive threshold values is often encountered in this context [28]. Using a relative threshold of 41% of SUV_max_ for 106 patients with peripheral T-cell lymphoma, Cottereau and colleagues demonstrated that baseline MTV is a relevant risk factor [22]. Kanoun and colleagues showed that MTV calculation using a fixed threshold of SUV 2.5 gave a higher volume than a relative limit of 41% of SUV_max_ [21]. In their cohort of 140 DLBCL patients, Kim and colleagues showed that a TLG calculated with 50% of the SUV_max_ has the highest prognostic accuracy when a relative threshold is used [26]. This is contrary to our results that indicate that a fixed threshold has a higher predictive value than a relative threshold. Furthermore, in a group of 121 patients, Tutino and co-workers observed that with a fixed threshold of SUV_4.0_ MTV-calculation is less dependent on the reviewer and can be reproduced better than calculation using a relative threshold of SUV_41%_ [29]. This is in line with our observation that MTV and TLG using a fixed cut-off of 4.0 may be slightly superior in terms of predicting PET-2 positivity. Here we have observed that MTV4.0 works comparably well in a cohort in which no SUV standardization between participating PET centers has been performed.

Due to the limited number of survival events, our analyses were restricted to PET-2 positivity and a further investigation of the influence on long-term efficacy in terms of progression-free survival is pending. However, as the prognostic influence of PET-2 on progression-free survival has been demonstrated for the HD16 trial [6], PET-2 could be regarded as a surrogate for longer-term efficacy. In order to further improve response prediction and risk-adapted individualization of therapy, additional risk factors are needed. Such risk factors might include but need not be restricted to the use of MTV and TLG, possibly in combination with PET-2. New biomarkers such as thymus and activation-regulated chemokine or cell free DNA would be well worth further investigation for individual tailoring of treatment in patients with Hodgkin lymphoma [30].

## Conclusion

MTV and TLG show predictive value after two cycles of ABVD in early-stage favorable Hodgkin lymphoma. When determining MTV and TLG, due to higher reproducibility and a slight advantage over a relative threshold, we favor a fixed cut-off of SUV 4.0.

However, it remains to be shown whether these factors can have a useful impact on prognosis when applied in combination with PET-2 assessment and other biomarkers in early stage Hodgkin lymphoma.

## Data Availability

From the corresponding author on request.

## Abbreviations

FDG: 18F -fluorodeoxyglucose
PET: Positron emission tomography
CT: Computed tomography
MTV: Metabolic tumor volume
TLG: Total lesion glycolysis
AUC: Area under the curve
SUV: Standardized uptake value
ABVD: Adriamycin, Bleomycin, Vinblastin und Dacarbazin
Gy: Gray
GHSG: German Hodgkin Study Group
PET-0: Baseline PET
PET-2: PET after 2 cycles of chemotherapy
VOI: Volume of interest
ROC: Receiver operating characteristic
eBEACOPP: Bleomycin, Etoposide, Doxorubicin, Cyclophosphamide, Vincristine, Procarbazine and Prednisone in escalated doses
DLBCL: Diffuse large B cell lymphoma
R-CHOP: Rituximab, Clophosphamid; Doxorubicin; Vincristin; Prednisone
